# Acetaminophen Metabolites on Presentation Following an Acute Acetaminophen Overdose (ATOM‐7)

**DOI:** 10.1002/cpt.2888

**Published:** 2023-04-06

**Authors:** Angela L. Chiew, Geoffrey K. Isbister, Paul Stathakis, Katherine Z. Isoardi, Colin Page, Kirsty Ress, Betty S.H. Chan, Nicholas A. Buckley

**Affiliations:** ^1^ Department of Clinical Toxicology Prince of Wales Hospital Randwick New South Wales Australia; ^2^ Faculty of Medicine The University of New South Wales Sydney New South Wales Australia; ^3^ New South Wales Poisons Information Centre Sydney Children's Hospital Sydney New South Wales Australia; ^4^ Department of Clinical Toxicology and Pharmacology, Calvary Mater Newcastle and School of Medical Practice University of Newcastle Callaghan New South Wales Australia; ^5^ NSW Health Pathology Prince of Wales Hospital Randwick New South Wales Australia; ^6^ Clinical Toxicology Unit Princess Alexandra Hospital Brisbane Queensland Australia; ^7^ Queensland Poisons Information Centre Queensland Children's Hospital Brisbane Queensland Australia; ^8^ Discipline of Biomedical Informatics and Digital Health The University of Sydney Sydney New South Wales Australia

## Abstract

Acetaminophen (APAP) is commonly taken in overdose and can cause acute liver injury via the toxic metabolite NAPQI formed by cytochrome (CYP) P450 pathway. We aimed to evaluate the concentrations of APAP metabolites on presentation following an acute APAP poisoning and whether these predicted the subsequent onset of hepatotoxicity (peak alanine aminotransferase > 1,000 U/L). The Australian Toxicology Monitoring (ATOM) study is a prospective observational study, recruiting via two poison information centers and four toxicology units. Patients following an acute APAP ingestion presenting < 24 hours post‐ingestion were recruited. Initial samples were analyzed for APAP metabolites, those measured were the nontoxic glucuronide (APAP‐Glu) and sulfate (APAP‐Sul) conjugates and NAPQI (toxic metabolite) conjugates APAP‐cysteine (APAP‐Cys) and APAP‐mercapturate (APAP‐Mer). The primary outcome was hepatotoxicity. In this study, 200 patients were included, with a median ingested dose of 20 g, 191 received acetylcysteine at median time of 5.8 hours post‐ingestion. Twenty‐six patients developed hepatotoxicity, one had hepatotoxicity on arrival (excluded from analysis). Those who developed hepatotoxicity had significantly higher total CYP metabolite concentrations: (36.8 μmol/L interquartile range (IQR): 27.8–51.7 vs. 10.8 μmol/L IQR: 6.9–19.5) and these were a greater proportion of total metabolites (5.4%, IQR: 3.8–7.7) vs. 1.7%, IQR: 1.3–2.6, *P* < 0.001)]. Furthermore, those who developed hepatotoxicity had lower APAP‐Sul concentrations (49.1 μmol/L, IQR: 24.7–72.2 vs. 78.7 μmol/L, IQR: 53.6–116.4) and lower percentage of APAP‐Sul (6.3%, IQR: 4.6–10.9 vs. 13.1%, IQR, 9.1–20.8, *P* < 0.001)]. This study found that those who developed hepatotoxicity had higher APAP metabolites derived from CYP pathway and lower sulfation metabolite on presentation. APAP metabolites may be utilized in the future to identify patients who could benefit from increased acetylcysteine or newer adjunct or research therapies.

**Study Highlights**

**WHAT IS THE CURRENT KNOWLEDGE ON THE TOPIC?**

Acetaminophen (APAP) is a common cause of drug‐induced liver injury. This occurs because of a toxic metabolite which is formed by the cytochrome P450 pathway. A previous study has shown that the cytochrome P450 pathway metabolites are sensitive for predicting liver injury (doubling in alanine aminotransferase (ALT)) on presentation.

**WHAT QUESTION DID THIS STUDY ADDRESS?**

Do APAP metabolites on presentation following an acute APAP poisoning predict which patients will develop hepatotoxicity (ALT > 1,000 U/L)?

**WHAT DOES THIS STUDY ADD TO OUR KNOWLEDGE?**

This study offers further insight into the toxicokinetic of APAP in overdose. Patients who developed liver injury had higher APAP metabolites derived from cytochrome P450 pathway that causes toxicity and lower sulfation metabolite on presentation.

**HOW MIGHT THIS CHANGE CLINICAL PHARMACOLOGY OR TRANSLATIONAL SCIENCE?**

The absolute concentrations of cytochrome P450 pathway APAP metabolites and combination of metabolites were good early predictors of liver injury on presentation. Cytochrome P450 and/or sulfate metabolites may allow for early recognition of high‐risk patients and possibly improve treatment algorithms or the use of adjunct treatments in these patients.

Acetaminophen (APAP) is a commonly utilized analgesic agent and in recommended doses is safe in healthy individuals. Due to its wide availability, APAP is one of the most common medications leading to hospital presentations and admissions following deliberate self‐poisoning and accidental overdose in many countries.^1^, ^2^ APAP toxicity is the leading cause of acute liver failure in North America, Europe, and Australia.^3^, ^4^ The mainstay of treatment is the antidote acetylcysteine. Its use in acute APAP overdose is guided by the history of ingestion, APAP concentration, and liver enzymes. Most acute APAP ingestions requiring treatment present within 8 hours of ingestion and are at low risk of liver injury.^5^ However, there are multiple reports of liver injury despite treatment within 8 hours of ingestion and liver injury despite an initial APAP concentration below the nomogram line.^6^, ^7^, ^8^ New biomarkers, including APAP metabolites, may improve identification of these patients for clinical and research purposes and provide insights into reasons for treatment “failure.”

APAP is mainly metabolized into two non‐toxic major metabolites, sulfate (APAP‐Sul) and glucuronide (APAP‐Glu) conjugates, which account for 30% and 55% of APAP metabolites excreted in urine.^9^ A highly reactive toxic metabolite, N‐acetyl‐p‐benzoquinone imine (NAPQI), is formed by cytochrome (CYP) P450 2E1 and 3A4. The small amounts of NAPQI produced after therapeutic doses of APAP are detoxified by glutathione dependent reactions to two nontoxic metabolites, mercapturic acid (APAP‐Mer) and cysteine conjugates (APAP‐Cys). In therapeutic doses, these 2 metabolites are excreted at ~ 4% (as a fraction of the parent dose) each with over 80% excreted in the urine in the first 12 hours following ingestion.^9^, ^10^ NAPQI is responsible for the hepatocellular injury that occurs with APAP toxicity. In overdose, glutathione can become depleted, and NAPQI can then bind to sulfhydryl groups in cellular proteins. This may lead to oxidative stress, mitochondrial injury, hepatocyte necrosis, and acute liver failure. The protein binding of NAPQI results in APAP protein adducts that can be quantified by measurement of APAP‐Cys that is released from the protein fraction of serum or plasma following protease enzyme treatment.^11^ This is a distinct pool of APAP‐Cys separate to the *in vivo* glutathione‐derived metabolite that is present in the nonprotein fraction of the circulation. If glutathione is depleted, NAPQI can no longer be detoxified, and it covalently binds to critical cellular proteins.^12^ APAP metabolites are detectable in plasma from healthy volunteers after therapeutic doses and in patients following an overdose.^13^, ^14^, ^15^, ^16^ They rapidly increase after ingestion of a therapeutic dose, with APAP‐Glu having a higher concentration than the parent drug from 1–2 hours after ingestion.^9^ Studies have shown that CYP metabolites and metabolite ratios are sensitive for predicting liver injury (doubling in alanine aminotransferase (ALT)) on presentation.^13^ Reliable prediction of those at increased risk is needed to facilitate research into better treatment pathways, such as selective use of increased acetylcysteine dosing and adjunct therapies, such as fomepizole.

The object of this study was to evaluate the concentrations of the APAP metabolites in acute APAP poisoning on presentation within 24 hours of ingestion and examine the ability of APAP metabolites or their ratios to other measures to predict which patients will subsequently develop acute liver injury.

## METHODS

### Design and setting

This study was nested within the Australian Toxicology Monitoring (ATOM) Collaboration, which consists of prospective observational studies to investigate various drugs and toxins in overdose.^17^, ^18^ ATOM is a multicenter collaboration comprising four toxicology units in New South Wales (NSW) and Queensland (QLD), Australia, and patients recruited through calls to the NSW and QLD Poisons Information Centres (PICs). The ATOM APAP study collected clinical data and at least 3 serum samples in the first 24 hours of admission at > 4 hours post‐ingestion or the time of admission, 4 hours later, and 1–2 hours before completion of the 20–21‐hour treatment protocol with acetylcysteine. If available, serum samples collected for patient management were also analyzed. This study aimed to examine the utility of the initial metabolite concentration and hence only utilized the initial metabolite concentrations (subsequent papers will examine the pharmacokinetics/pharmacodynamics of the acetaminophen metabolites). The ATOM study received ethical approval from Human Research and Ethics Committees in NSW and QLD that covered all involved institutions and patients provided informed consent for participation in the study.

### Selection of participants

The ATOM study APAP project, recruits patients (≥ 14 years) from two sources. First, they were recruited from two toxicology units (Prince of Wales and Calvary Mater Newcastle), if they were assessed for APAP ingestion regardless of intent or preparation (immediate or modified release). Second, higher‐risk patients were also recruited from the NSW and QLD PIC (via telephone calls for clinical advice) and two further toxicology units (Royal Prince Alfred Hospital and Princess Alexandra). These patients were included if they met any of the following criteria: any acute ingestion (over a period of < 8 hours) of immediate release acetaminophen ≥ 35 g, or any acute ingestion of modified‐release APAP ≥ 10 g, or ≥ 200 mg/kg (whichever was less), or any patients with an ALT ≥ 500 U/L (10 times the upper limit of normal) following an APAP ingestion (regardless of intent or preparation). The study recruited from January 2013 until December 2016 with recruitment commencing at varying times dependent on ethics approval. In this study, a subset of these patients was included. The inclusion criteria were acute ingestions of immediate or modified‐release acetaminophen presenting within 24 hours of ingestion with a study serum sample taken with the initial acetaminophen concentration that was available for metabolite analysis. Acute ingestions were defined as an ingestion occurring over less than an 8‐hour period.^19^ Data from some of these patients has been reported previously^17^, ^18^, ^20^ but not metabolite results. The Prince of Wales and Calvary Mater Newcastle Toxicology units each manage around 1,000 toxicology patients annually. The NSW and QLD PIC receives ~ 67,000 and 40,000 calls per year from residents in NSW and QLD, respectively.^21^

### Methods and measurements

Clinical data were collected on a preformatted clinical datasheet and from medical records. Data collected included demographic information, overdose exposure (time (taken from the earliest possible time of ingestion) and dose ingested), and co‐ingestions, including ethanol, laboratory results, treatments, and outcomes. In Australia, APAP is available as immediate release (IR) and modified release (MR) formulations. Each MR APAP tablet contains 665 mg of APAP of which 69% is slow‐release and 31% IR APAP in a bilayer tablet. There were some patients (*n* = 19) for whom an ALT was not recorded at the completion of acetylcysteine treatment or 24 hours post‐ingestion for those not requiring acetylcysteine. These patients had no clinical symptoms when discharged and were assumed to have not developed acute liver injury.

### Analytical method for APAP metabolites

Serum samples were collected and frozen to −80°C for batch analysis. APAP and its metabolite concentrations were measured with an AB SCIEX Triple QuadTM 5,500 liquid chromatography with tandem mass spectrometry system (LC‐MS/MS; derived from An *et al*.^6^) using both positive ionization mode for APAP, and APAP‐d4 (internal standard), and negative ionization mode for APAP‐Glu, APAP‐Sul, APAP‐Cys, APAP‐Mer, and APAP‐Glcd3 (internal standard).^6^ Intra‐ and interassay variations were assessed from the quality control samples, utilizing low and high concentrations for all metabolites. Intra‐assay variation ranged from 3.6% to 9.5%. Interassay variation ranged from 4.9% to 11.2%. The lower limit of quantitation of the assay was determined by the lowest quality control concentration measurable with a coefficient of variability of < 20%. The lower limit of quantitation for the assay for APAP, APAP‐Glu, and APAP‐Sul was 1.3 μmol/L, 0.6 μmol/L, and 0.9 μmol/L, respectively, and for APAP‐Cys and APAP‐Mer was 0.02 μmol/L. The laboratory technician was blinded to clinical histories and outcomes. The LC–MS/MS analysis was performed at South‐Eastern Area Laboratory Services, Prince of Wales Hospital, Sydney, Australia.

#### Clinical outcomes

Acute liver injury was defined as a peak ALT > 1,000 U/L (hepatotoxicity).^22^ We also examined a peak ALT > 100 U/L (the UK criteria for the use of additional acetylcysteine beyond the standard 20–21‐hour course).^23^ ALT is a continuous measure, however, these dichotomous outcomes were chosen as they are commonly utilized clinically and in research. We also recorded complications, such as coagulopathy (defined as an international normalized ratio (INR) > 2.0), severe acute kidney injury (AKIN classification stage 3: serum creatinine rise of ≥ 3× baseline or a rise of ≥ 1.5 baseline to > 354 μmol/L, a urine output < 0.3 mL/kg/h for ≥ 24 hours, or anuria for ≥ 12 hours),^24^ liver transplant, and death.

#### Statistical analysis for prediction of liver injury

All data are presented as median and range or interquartile range (IQR; non‐normally distributed data). We compared the APAP concentration and initial metabolites in those with and without acute liver injury using the Mann–Whitney *U* test, as the data were not normally or log‐normally distributed.^13^ All LC‐MS/MS data were transformed from mass to molar concentrations before analyses were performed. Predictive performance was compared using the area under the receiver‐operating‐characteristic curve (AUC‐ROC). The analysis was repeated excluding those who had acetylcysteine commenced prior to metabolite measurement. Associations between predictive variables was measured with Pearson's or Spearman's coefficient, depending on whether a nonlinear relationship was observed. A *P* value of < 0.05 was considered statistically significant. All analysis was performed using GraphPad PRISM software version 8.0.2.

For ROC analysis, only those patients with an initial ALT ≤ 1,000 U/L at the time of metabolite measurement were included. A further analysis to predict an ALT > 100 U/L was performed with those patients with an initial ALT ≤ 100 U/L.

To compare risk based on APAP concentrations between patients at different timepoints, the APAP ratio was calculated which utilized the initial APAP concentration obtained between 4 and 24 hours post‐ingestion.^22^, ^25^

$$\text{Acetaminophen ratio}=\frac{\text{First acetaminophen concentration taken}\geq 4\;h\;\text{post ingestion}\;\left(\mathrm{but}\leq 24\;h\right)}{\text{Acetaminophen concentration}\;\mathrm{on}\;\text{the}\;\left(150\;\mathrm{mg}/L\;\mathrm{at}\;4\;h\right)\;\text{standard nomogram line}\;\mathrm{at}\;\text{that time point}}$$

Metabolites were all analyzed in molar units to facilitate comparisons of ratios. For each patient the following was calculated on initial serum sample:

- Sum of all metabolites and total CYP (APAP‐Cys + APAP‐Mer) metabolites.
- Percentages for each metabolite and total CYP metabolites (% CYP metabolites).
- APAP‐Cys/APAP‐Sul ratio (identified as best predictor in previous study).^13^

We also explored if prediction was further improved by using multiplication products of combinations of currently used predictors (ALT and APAP ratio) with metabolites (i.e., APAP‐Cys and APAP‐Cys/APAP‐Sul).

Hospital APAP concentration analysis that was reported as lower than the laboratory limit of detection (range of detection between 1 and 10 mg/L) were analyzed as half this limit (*n* = 5). If the lower limit of detection was not reported and an APAP concentration was reported as “not detected,” a lower limit of detection of 5 mg/L was used when calculating the APAP ratio.

## RESULTS

Of the 318 patients recruited, 249 had an initial serum sample available for analysis for APAP metabolite concentrations. We then excluded 33 patients with repeated supratherapeutic ingestion and 16 acute ingestions presenting > 24 hours post‐ingestion leaving 200 who met inclusion criteria. This cohort included 140 (70%) women and had a median age of 25 years (IQR: 18–39 years) and a median weight of 65 kg (IQR: 58–80 kg, *n* = 198). The majority were IR ingestions (*n* = 152, 76%), 37 were MR, and 11 were a combination of products. The median ingested dose was 20 g (IQR: 10–41 g, *n* = 199). Demographic data, treatments, and outcomes according to preparation ingested are shown in **Table** **1**.

**Table 1:** Demographic data, treatment, and outcome of patients

	All patients (*n* = 200)	IR (*n* = 152)	MR^a^ (*n* = 48)
Females (%)	140 (70%)	109 (72%)	31 (65%)
Median age (years) (IQR)	25 (18–39)	24 (18–38)	27 (19–50)
Median weight (kg) (IQR)	65 (58–80) (*n* = 198)	65 (55–80) (*n* = 151)	68 (60–77) (*n* = 47)
Median dose ingested (g) (IQR)	20 (10–41) (*n* = 199)	20 (10–40) (*n* = 152)	31.9 (16.5–45.4) (*n* = 47)
Median dose ingested (g/kg) (IQR)	0.32 (0.17–0.60) (*n* = 197)	0.30 (0.16–0.57) (*n* = 151)	0.40 (0.23–0.69) (*n* = 46)
Co‐ingested ethanol (%)	40 (20%)	27 (18%)	13 (27%)
Median time to presentation (h) (IQR)	2.9 (1.8–7.5) (*n* = 200)	2.8 (1.7–7.0) (*n* = 152)	3.0 (2.0–9.0) (*n* = 48)
Received activated charcoal (%)	27 (14%)	18 (12%)	9 (19%)
Median time to activated charcoal (h)	2 (1.2–4.5)	1.9 (1.0–3.4)	3.5 (1.5–4.5)
ALT at presentation > 50 U/L	35 (18%)	23 (15%)	12 (25%)
Acetaminophen concentration above the nomogram and/or ALT >50 U/L in those presenting >8 h post ingestion	123^b^ (62%)	86 (57%)	37^b^ (77%)
Commenced on acetylcysteine	191 (96%)	144 (95%)	47 (98%)
Median time to acetylcysteine (h) (IQR)	5.8 (3.6–9.7)	5.8 (3.5–10.8) (*n* = 144)	5.2 (4.0–11.7) (*n* = 47)
Required prolonged treatment with acetylcysteine (i.e. >20–21 h)	72 (36%)	47 (31%)	25 (52%)
Peak ALT b/w >100 U/L and 1,000 U/L	16 (8%)	12 (8%)	4 (8%)
Peak ALT ≥1,000 U/L	26 (13%)	18 (12%)	8 (17%)

^a^ Includes patients who ingested MR acetaminophen (*n* = 36) or a combination of MR and IR (*n* = 11).

^b^ For MR ingestions includes patients with either the initial or repeat acetaminophen concentration above the nomogram line. Note this includes three patients with the first acetaminophen concentration below the nomogram line and the repeat concentration above and one further patient who crossed the nomogram line on the third acetaminophen concentration (measured 4 h apart).

Most patients 191 (96%) received acetylcysteine, either the 2 or 3 bag regimen (as per local protocols) of 300 mg/kg over 20–21 hours at a median time of 5.8 hours (IQR: 3.6–9.7 hours post‐ingestion). Of these, 37 patients were administered an increased dose of acetylcysteine, most commonly an increase in the final infusion from 100 mg/kg over 16 hours to 200 mg/kg over 16 hours and 72 required prolonged acetylcysteine treatment beyond the standard 20–21‐hour infusion. Forty‐two (21%) patients developed a peak ALT > 100 U/L, of which 26 developed hepatoxicity (13%). One patient received a liver transplant and one patient died (87 MALE died from respiratory failure secondary to aspiration 30 hours post‐ingestion). Fifteen developed an INR > 2.0 of which 5 had an INR > 5.0 and 4 developed severe acute kidney injury (AKIN classification stage 3).

### Acetaminophen metabolites on presentation

**Figure 1:**
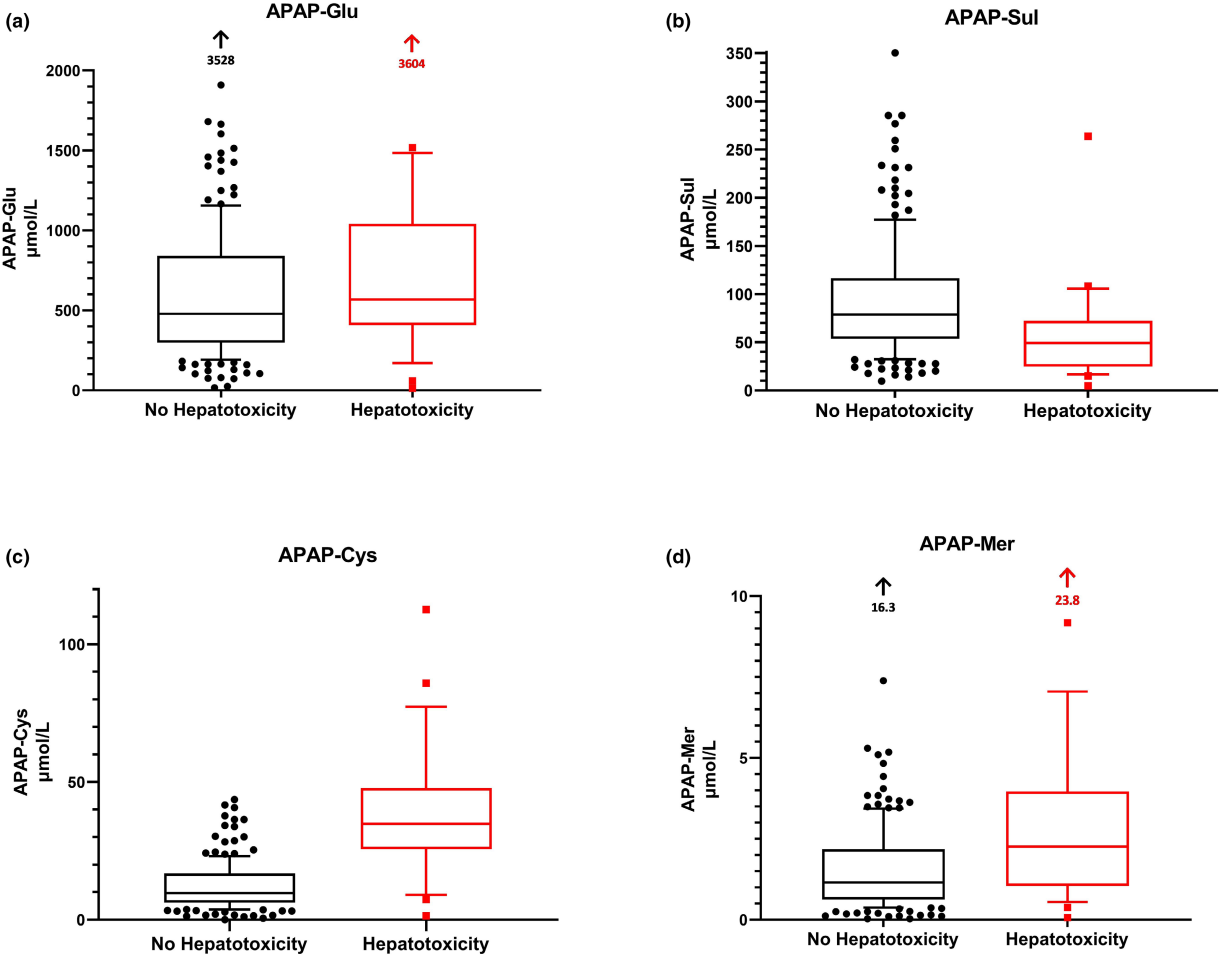
Box and whiskers plot of initial acetaminophen metabolite concentrations stratified according to outcome ALT > 1,000 U/L. (**a**) APAP‐Glu, (**b**) APAP‐Sul, (**c**) APAP‐Cys, (**d**) APAP‐Mer. Only including those patients with an initial ALT < 1,000 U/L. Box and whiskers plot the midline bar represents the median value, box represents the first and third quartile, bars the 10th and 90th centiles. ALT, alanine aminotransferase; APAP, acetaminophen; APAP‐Cys, acetaminophen‐cysteine; APAP‐Glu, acetaminophen‐glucuronide; APAP‐Mer, acetaminophen‐mercapturate; APAP‐Sul, acetaminophen‐sulfate.

APAP parent drug concentration measured by LC–MS/MS correlated significantly with the value from the clinical laboratory APAP assay. With a Pearson *r* value 0.92 (95% confidence interval: 0.89–0.94, *P* < 0.0001), and correlation coefficient (*R* ^2^) of 0.84 (*n* = 195) in only those with a detectable acetaminophen concentration; **Figure** **S1**). On presentation, APAP‐Glu was the metabolite with the highest concentration followed by APAP‐Sul, APAP‐Cys, and APAP‐Mer (**Table** **2**, **Figure** **1**). Acetylcysteine had already been commenced in 59 (29%) patients at the time of metabolite measurement.

A comparison of traditional biomarkers (i.e, ALT, aspartate aminotransferase, and APAP concentration) and APAP metabolites were made examining those who developed hepatotoxicity (**Table** **2**, **Figure** **1**) and an ALT > 100 U/L (**Table** **3**). Only those patients with an ALT < 1,000 U/L (*n* = 199) on arrival were utilized for the hepatotoxicity analysis and those with an ALT < 100 U/L for the ALT ≥ 100 U/L analysis (*n* = 179).

**Table 2:** Outcome hepatotoxicity, only those with ALT <1,000 U/L on presentation

Metabolites/Biomarker with initial acetaminophen concentration	No hepatotoxicity (median) (IQR, [range]) (*n*) (*n* = 174)	Hepatotoxicity (median) (IQR, [range]) (*n*) (*n* = 25)	Difference between medians (95% CI)	*P* value
APAP‐hospital lab (μmol/L)	702 (292–1,351, [3–5,614]) (*n* = 174)	1,106 (528–1,969, [33–3,423]) (*n* = 25)	404 (−25 to 655)	NS
ALT (U/L)	19 (14–28, [5–276]) (*n* = 172)	127 (49–221, [18–726]) (*n* = 25)	108 (53 to 141)	<0.0001
Acetaminophen ratio	1.1 (0.4–2.1, [0.0–10.3]) (*n* = 174)	3.6 (2.3–7.8, [0.3–27.6]) (*n* = 25)	2.5 (1.8 to 3.9)	<0.0001
Acetaminophen metabolite				
APAP‐LC/MS (μmol/L)	675 (291–1,196, [8–4,634]) (*n* = 174)	867 (402–1,460, [10–4,800] (*n* = 25)	192 (–123 to 464)	NS
APAP‐Glu (μmol/L)	479 (299–841, [16–3,529] (*n* = 174)	568 (409–1,041, [13–3,605] (*n* = 25)	89 (–55 to 264)	NS
APAP‐Sul (μmol/L)	79 (54–116, [10–350]) (*n* = 174)	49 (25–72, [5–264]) (*n* = 25)	–30 (–48 to [–15])	0.0002
APAP‐Cys (μmol/L)	9.7 (6.2–16.8, [0.0–43.6]) (*n* = 174)	34.8 (25.6–47.8, [1.4–112.6]) (*n* = 25)	25.1 (18.5 to 29.8)	<0.0001
APAP‐Mer (μmol/L)	1.2 (0.6–2.2, [0.03–16.32] (*n* = 174)	2.3 (1.1–4.0, [0.07–23.8]) (*n* = 25)	1.1 (0.29 to 1.5)	0.003
CYP total (μmol/L)	10.8 (6.9–19.5, [0.3–57.0]) (*n* = 174)	36.8 (27.8–51.7, [1.4–136.4]) (*n* = 25)	26.1 (19.1 to 31.6)	<0.0001
Total metabolites	589 (382–979, [27–3,862]) (*n* = 174)	654 (459–1,179, [19–3,845]) (*n* = 25)	64 (–79 to 262)	NS
% APAP‐Glu	85 (77–89, [23–95]) (*n* = 174)	89 (82–90, [67–94]) (*n* = 25)	3.9 (–0.09 to 5.7)	NS
% APAP‐Sul	13.1 (9.1–20.8, [2.8–67]) (*n* = 174)	6.3 (4.6–10.9, [2.7–24.7]) (*n* = 25)	–6.8 (–8.7 to [–3.4])	<0.0001
% CYP metabolites	1.7 (1.3–2.6, [0.1–18.2]) (*n* = 174)	5.4 (3.8–7.7, [1.1–14.5]) (*n* = 25)	3.7 (2.7 to 4.3)	<0.0001
Proposed risk stratification tools				
APAP‐Cys/APAP‐ Sul	0.12 (0.06–0.21, [0.0–1.7]) (*n* = 174)	0.83 (0.44–1.08, [0.1–2.2]) (*n* = 25)	0.7 (0.5 to 0.8)	<0.0001
ALT * APAP‐Cys/APAP‐Sul	2.2 (1.1–4.5, [0.0–75.6]) (*n* = 172)	116 (23.7–212, [1.9–997]) (*n* = 25)	114 (86 to 144)	<0.0001
Acetaminophen ratio*APAP‐Cys	10 (2–27, [0.0–352]) (*n* = 174)	118 (33–289, [2–2,370]) (*n* = 25)	108 (75 to 178)	<0.0001
Acetaminophen ratio*APAP‐Cys*ALT	186 (50–528, [0.0–12,194]) (*n* = 172)	15,543 (1,887–66,184, [509–378,805]) (*n* = 25)	15,357 (6,267 to 18,878)	<0.0001

NS, not significant.

**Table 3:** Outcome ALT ≥100 U/L, only those whose ALT at presentation was <100 U/L

Metabolites/Biomarker	No acute liver injury (peak ALT < 100 U/L) (median) (IQR, [range], *n*) (*n* = 158)	(Peak ALT ≥ 100 U/L) (median) (IQR, [range], *n*) (*n* = 21)	Difference between medians (95% CI)	*P* value
APAP‐hospital lab (μmol/L)	686 (271–1,357, [3.3–5,614]) (*n* = 158)	940 (504–2,815, [301–3,856]) (*n* = 21)	254 (50 to 747)	0.030
ALT (U/L)	18 (14–25, [5–90]) (*n* = 156)	40 (19–57, [14–78]) (*n* = 21)	22 (6 to 26)	<0.0001
Acetaminophen Ratio	1.0 (0.4–1.9, [0.0–10.3]) (*n* = 158)	3.3 (11.8–6.0, [0.7–27.6]) (*n* = 21)	2.3 (1.3 to 3.0)	<0.0001
Acetaminophen metabolite				
APAP‐LC/MS (μmol/L)	685 (296–1,228, [7.7–4,634]) (*n* = 158)	894 (493–2,140, [266–4,800]) (*n* = 21)	209 (−29 to 597)	NS
APAP‐Glu (μmol/L)	472 (314–834, [16–3,529]) (*n* = 158)	629 (425–990, [209–1,680]) (*n* = 21)	157 (−38 to 312)	NS
APAP‐Sul (μmol/L)	79 (56–115, [9.7–350]) (*n* = 158)	60 (37–95, [15–264]) (*n* = 21)	−19 (−38 to 2.6)	NS
APAP‐Cys (μmol/L)	9.4 (5.9–15.8, [0.0–41.7]) (*n* = 158)	30.1 (19.6–39.3, [7.2–85.9]) (*n* = 21)	20.9 (14.0 to 25.2)	<0.0001
APAP‐Mer (μmol/L)	1.1 (0.6–2.1, [0.0–16.3]) (*n* = 158)	2.7 (1.5–3.6, [0.4–9.2]) (*n* = 21)	1.6 (0.7 to 2.0)	<0.0001
CYP total (μmol/L)	10.5 (6.7–18.0, [0.3–57]) (*n* = 158)	33.5 (21.3–43.2, [7.5–89.4]) (*n* = 21)	23 (15 to 27)	<0.0001
Total metabolites (μmol/L)	587 (390–976, [27–3,862]) (*n* = 158)	687 (522–1,179, [254–1,815]) (*n* = 21)	99 (−47 to 321)	NS
% APAP‐Glu	85 (77–89, [23–95]) (*n* = 158)	87 (82–91, [58–93]) (*n* = 21)	2.3 (−0.45 to 6.1)	NS
% APAP‐Sul	13.2 (9.3–21.3, [2.8–67]) (*n* = 158)	7.5 (5.4–11.2, [2.8–40.2]) (*n* = 21)	−5.7 (−8.4 to [−2.6])	0.0002
% CYP metabolites	1.6 (1.3–2.4, [0.1–18.2]) (*n* = 158)	4.1 (2.6–6.8, [1.1–10.3]) (*n* = 21)	2.5 (1.6 to 3.7)	<0.0001
Proposed risk stratification tools				
APAP‐Cys/APAP‐Sul	0.12 (0.06–0.19, [0.0–1.4]) (*n* = 158)	0.54 (0.25–0.98, [0.1–2.0]) (*n* = 21)	0.43 (0.24 to 0.65)	<0.0001
ALT*APAP‐Cys/APAP‐Sul	2.0 (1.0–3.8, [0.0–68.8]) (*n* = 156)	17.5 (5.8–38.7, [1.2–94.2]) (*n* = 21)	15.5 (5.8 to 24.2)	<0.0001
Acetaminophen ratio*APAP‐Cys	8 (2–23, [0–1,004]) (*n* = 158)	80 (36–221, [9–4,459]) (*n* = 21)	72 (37 to 109)	<0.0001
Acetaminophen ratio*APAP‐Cys*ALT	156 (47–387, [0–17,059]) (*n* = 156)	3,202 (807–7,582, [286–347,810]) (*n* = 21)	3,046 (1,325 to 3,345)	<0.0001

NS, not significant. [Correction added on 03 May 2023, after first online publication: In Table 3, the n value of third column heading ((Peak ALT ≥ 100 U/L) (median) (IQR, [range], n) (n = 21)) has been corrected in this version.]

**Figure 2:**
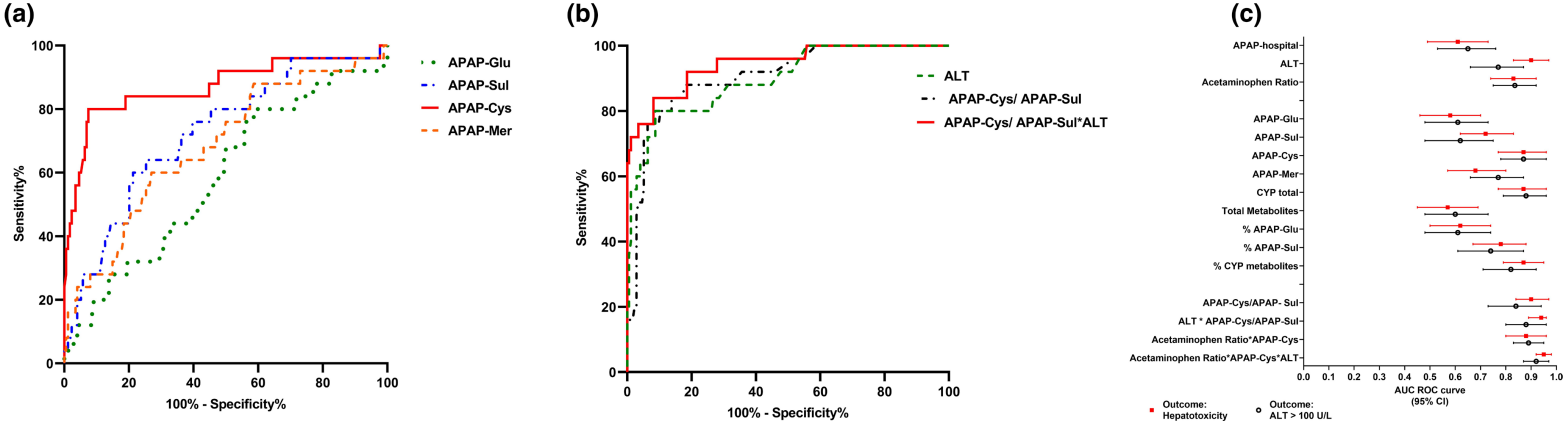
ROC analysis of the ability of APAP metabolite concentration, ALT and metabolite ratios to predict hepatotoxicity. ROC analysis of the ability of (**a**) initial APAP metabolite concentrations (**b**) ALT, APAP‐Cys/APAP‐Sul and APAP‐Cys/APAP‐Sul*ALT (**c**) Forest plot of AUC‐ROC to predict hepatotoxicity (ALT >1,000 U/L) (squares) and ALT >100 U/L (open circles). ALT, alanine aminotransferase; APAP, acetaminophen; APAP‐Cys, acetaminophen‐cysteine; APAP‐Glu, acetaminophen‐glucuronide; APAP‐Mer, acetaminophen‐mercapturate; APAP‐Sul, acetaminophen‐sulfate; AUC, area under the curve; CI, confidence interval; ROC, receiver operating characteristic curve.

There were significantly higher CYP metabolite concentrations (APAP‐Cys and APAP‐Mer) and ALT (**Tables** **2**, **3**, **Figure** **1**) on presentation in those who developed hepatotoxicity or an ALT > 100 U/L. There was a significantly lower concentration of APAP‐Sul on presentation in those developing hepatoxicity (**Table** **2**, **Figure** **1**). There were also significant differences in the percentages of APAP‐Sul and CYP metabolites (i.e., lower APAP‐Sul % and higher CYP metabolite % in those that developed liver injury; **Tables** **2**, **3**). There was a significantly higher APAP‐Cys/APAP‐Sul ratio in those that developed liver injury. The highest AUC‐ROC curve to predict hepatotoxicity on presentation was for the APAP‐Cys/APAP‐Sul ratio and ALT (**Figure** **2**). The results remained similar when those who had acetylcysteine prior to metabolites measured were excluded (**Table** **S1**).

### Combination of biomarkers

Various combinations of traditional liver biomarkers (i.e., ALT), APAP concentration (i.e., APAP ratio), and APAP metabolites were also examined based on combining the best performing markers (**Tables** **2**, **3**). The multiplication of APAP ratio*APAP‐Cys*ALT had the highest AUC‐ROC curve to predict hepatoxicity: (0.95, 95% confidence interval: 0.92–0.98; **Figure** 2).

### Metabolite correlation

**Figure 3:**
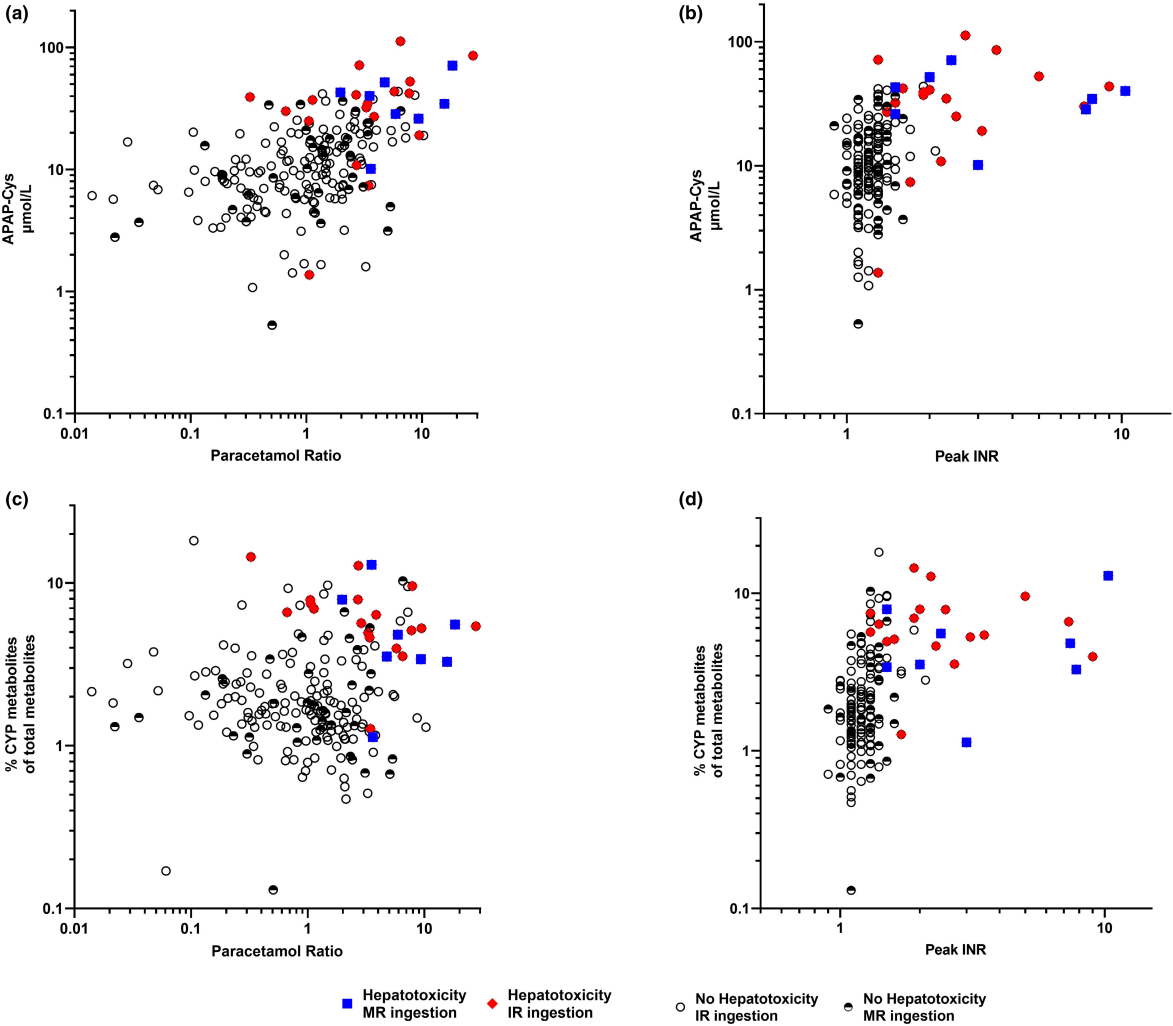
Correlation among: (**a**) APAP‐Cys and acetaminophen ratio (*n* = 200). (**b**) APAP‐Cys and peak INR (*n* = 165). (**c**) Percentage of CYP metabolites of total metabolites and paracetamol ratio (*n* = 200). (**d**) Percentage of CYP metabolites of total metabolites and peak INR (*n* = 165). APAP, acetaminophen; APAP‐Cys, acetaminophen‐cysteine; INR, international normalized ratio; IR, immediate release; MR, modified release.

We examined for correlations between metabolite biomarkers and clinical factors (**Table** **4**, **Figure** **3**). Specifically, we examined for correlations among APAP‐Cys, APAP‐Sul, APAP‐Cys/APAP‐Sul, and CYP metabolites/total metabolites on presentation with age, dose ingested (mg/kg), APAP ratio, peak ALT, and peak INR. There was no correlation with age and CYP metabolites or their ratios. The strongest correlation was seen with peak INR and time post‐ingestion and the CYP metabolites or their ratios. However, the correlation was only moderate (Spearman R 0.50; **Table** **4**, **Figure** **3**).

**Table 4:** Correlation (*R* value) between APAP‐Cys, APAP‐Sul, % CYP metabolites (CYP metabolites/total metabolites) and risk factors for liver injury and outcomes

	APAP‐Cys	APAP‐Sul	% CYP metabolites	APAP‐Cys/APAP‐Sul
	*R* value (95% CI)	*R* value (95% CI)	*R* value (95% CI)	*R* value (95% CI)
	(*n* = 200)	(*n* = 200)	(*n* = 200)	(*n* = 200)
Risk factors				
Dose (mg/kg)^b^ (*n* = 197)	0.26 (0.13 to 0.39)	0.19 (0.05 to 0.32)	−0.08 (−0.22 to 0.07)	0.09 (−0.05 to 0.23)
Age^a^ (*n* = 200)	0.08 (−0.07 to 0.22)	0.29 (0.15 to 0.41)	−0.02 (−0.17 to 0.12)	−0.09 (−0.23 to 0.05)
Time post ingestion^a^ (*n* = 200)	0.45 (0.32 to 0.55)	−0.28 (−0.41 to −0.14)	0.49 (0.38 to 0.60)	0.49 (0.38 to 0.59)
Acetaminophen ratio^b^ (*n* = 200)	0.61 (0.52 to 0.69)	0.004 (−0.13 to 0.14)	0.19 (0.06 to 0.32)	0.40 (0.27 to 0.51)
Outcomes				
Peak ALT^a^ (*n* = 199)	0.39 (0.26 to 0.50)	−0.14 (−0.28 to 0.00)	0.36 (0.23 to 0.48)	0.34 (0.21 to 0.46)
Peak INR^a^ (*n* = 165)	0.47 (0.34 to 0.59)	−0.27 (−0.41 to −0.12)	0.50 (0.38 to 0.61)	0.38 (0.51 to 0.62)

^a^ Spearman *R* correlation.

^b^ Pearson *R* correlation.

## DISCUSSION

This study of 200 acute APAP overdoses offers further insight into the toxicokinetics of APAP in overdose. It demonstrated that those who developed acute liver injury had a higher concentration and percentage of CYP metabolites on presentation compared with those who did not. Metabolite concentrations may be a useful early biomarker of liver injury risk and potentially identify those who might benefit most from inhibitors of CYP metabolite production, such as fomepizole or other enhanced treatments.

Acetylcysteine replenishes glutathione that detoxifies the toxic metabolite NAPQI.^10^, ^26^ Although it is an effective antidote, it has therapeutic limitations, with less effectiveness in patients who present following a massive overdose, MR overdose, and delayed APAP presentations (>8 hours post‐ingestion).^18^, ^20^, ^25^, ^27^ Fomepizole has been proposed as an adjunct therapy. It is a CYP2E1 inhibitor, so it can decrease the formation of NAPQI and augment acetylcysteine treatment. However, the evidence for its use is limited to case reports and small series in high‐risk APAP poisonings.^27^, ^28^, ^29^ A crossover study of 5 healthy volunteers receiving fomepizole just prior to a single dose of 80 mg/kg of APAP^30^ showed fomepizole led to significantly lower concentrations of the CYP metabolites detected both in serum and urine. However, it is unclear what effect fomepizole has when administered many hours after an overdose, when CYP concentrations may already be elevated. In our study, we found a moderate correlation with time post‐ingestion and total concentration of CYP metabolites and a higher percentage of CYP metabolites with time. Hence, early administration of fomepizole is likely required for maximum therapeutic effect but this might be informed by CYP metabolite concentrations.

This study gives further insight into the concentration of CYP metabolites in those with a high APAP ratio on presentation. Those with a high initial APAP concentration (> 300 mg/L at 4 hours nomogram line) are at an increased risk of liver injury despite early acetylcysteine administration.^7^, ^8^, ^20^ Administration of higher doses of acetylcysteine has been shown to decrease this risk because the standard 300 mg/kg i.v. acetylcysteine regimen over 20–21 hours is inadequate for some patients with “massive” ingestions.^6^, ^20^, ^31^ However, not all patients with a high initial APAP concentration develop liver injury. The variation in those who developed liver injury may be explained by examining the CYP metabolite concentrations in those with high APAP ratios. In this study, some patients with a high initial APAP ratio > 2 had low concentrations of APAP‐Cys (**Figure** **3** **a**), whereas those who developed hepatotoxicity had higher concentrations (**Table** **2**). Unfortunately, we cannot yet measure CYP metabolites clinically and instead some guidelines administer an increased dose of acetylcysteine to all patients with a high initial APAP ratio.^32^ This finding has important implications when interpreting case reports and case series in which fomepizole is utilized early to decrease the production of NAPQI.^28^, ^29^ Without measuring CYP metabolites we cannot know whether fomepizole was of benefit. Hence, in future case reports and studies of fomepizole, it is important that metabolites are measured to determine if the patient had high CYP metabolites and the effect of fomepizole. Hence, measuring CYP metabolites on presentation may be helpful in identifying those patients who would benefit from increased acetylcysteine and adjuvant fomepizole.

We found no significant difference in the proportion of APAP‐Glu metabolites in those who did or did not develop liver injury. However, there were difference in APAP‐Sul and CYP metabolites. Glucuronidation, is a non‐saturable pathway, compared with sulfation, which is considered a high‐affinity, low‐capacity saturable process.^33^, ^34^ With sulfation it is often said to be “saturated” at therapeutic doses and hence not an important pathway.^35^ Sulfation of APAP is mainly catalyzed by sulfotransferase (SULT)1A1 and SULT1A3/4,^36^ with inorganic sulfate providing the source of sulfate for SULT. There are genetic polymorphisms of SULTs, which are associated with variable sulfation activity.^36^ Serum inorganic sulfate concentrations are typically 0.3–0.4 mmol^37^ and accounts for 90–95% of the total body sulfate.^38^ A low‐sulfate diet is a risk factor for reduced SULT activity.^39^ Hence, possible reasons for lower sulfation capacity in some individuals may include genetic polymorphism of SULT and/or limited sulfate availability.

Sulfate is consumed after APAP ingestion, which limits APAP sulfation capacity.^40^ It has been proposed that insufficient sulfation and sulfate depletion by APAP could shift the biotransformation of APAP from the sulfation pathway to alternative pathways.^40^ Alternatively, both sulfate and glutathione are consumable and have finite stores. Sulfate is the final oxidized product of cysteine and cysteine is one of the three amino acids that form glutathione. This results in a mutually competitive relationship between sulfation of APAP and glutathione detoxification. Hence, reduced metabolism by sulfation may be an indicator or marker of depletion of sulfate and may merely represent an association with glutathione depletion. It has been proposed that serum sulfate may be a potential biomarker for early identification of those at risk of liver injury.^40^ Further research is required into the role of sulfation, whether serum sulfate concentrations correlate with APAP‐Sul concentrations, and whether sulfate supplementation would improve metabolism in these patients.

In acute APAP poisonings, APAP‐Cys/APAP‐Sul detects those at risk of developing subsequent acute liver injury (defined as an increased serum ALT activity of 50% or more) with an AUC‐ROC 0.91 (0.83–0.98).^13^ We confirmed this association for hepatotoxicity with a similarly high AUC‐ROC. APAP‐Cys/APAP‐Sul reflects both an increase in APAP‐Cys production and/or a decrease in APAP‐Sul. These results may indicate it is the balance among glutathione depletion, CYP metabolism, and sulfation that is important. However, we found that a combination of markers performed even better to predict acute liver injury. These represent a combination of traditional liver biomarkers, APAP body load (e.g., APAP ratio), and APAP metabolites. APAP metabolites complement these traditional markers by offering potential refinement of risk stratification. This may help identify those patients who may benefit from newly proposed adjuvants to acetylcysteine, such as fomepizole.

Limitations of this study include its reliance on patient reporting of timing of ingestion and dose of APAP. However, generally, these aspects of clinical history are carefully recorded as they drive treatment decisions. Second, some patients had early initiation of acetylcysteine prior to the 4 hours APAP concentration on the treating doctor's discretion or local guidelines and the initial metabolites concentrations was post‐initiation of acetylcysteine. Acetylcysteine replenishes glutathione and is a substrate for sulfation.^41^ Hence, acetylcysteine treatment may increase sulfation and/or CYP metabolites in those who are glutathione and/or sulfate depleted. However, when these patients were excluded (**Table** **S1**) the results remained similar. Other limitations included the number of patients with hepatotoxicity and fulminant hepatic failure was low, particularly those with an initial ALT < 50 U/L who went on to develop acute liver injury. Hence, further studies with more patients are required to validate our findings and to determine if metabolites can aid in better prediction of outcomes, such as liver transplant or death. Some patients in this study with an initial non‐toxic patient's concentration did not have a repeat ALT at 24 hours. Although uncommon, some patients may have developed liver injury.

## CONCLUSION

This study found that absolute concentrations of CYP pathway APAP metabolites and combination of metabolites were good early predictors of liver injury on presentation. CYP and/or sulfate metabolites may allow for early recognition of high‐risk patients and possibly improve treatment algorithms or the use of adjunct treatments in these patients.

## FUNDING

This research was partially supported by an NHMRC Program Grant 1055176. Geoff Isbister is funded by an NHMRC Senior Research Fellowship ID1061041.

## CONFLICT OF INTEREST

The authors declared no competing interests for this work.

## AUTHOR CONTRIBUTIONS

A.L.C. and N.A.B. wrote the manuscript. A.L.C., N.A.B., C.P., and G.K.I. designed the research. All authors performed the research. A.L.C. and N.A.B. analyzed the data.

## Supporting information


Data S1
