# Supplementary material for: Acetaminophen Metabolites on Presentation Following an Acute Acetaminophen Overdose (ATOM‐7)
Source: Clin Pharmacol Ther. 2023 Apr 6;113(6):1304–14. doi: 10.1002/cpt.2888 (PMC10952325; doi:10.1002/cpt.2888)
Supplement: Supplementary file 1 — Data S1 [file CPT-113-1304-s001.pdf]

Supplementary Files

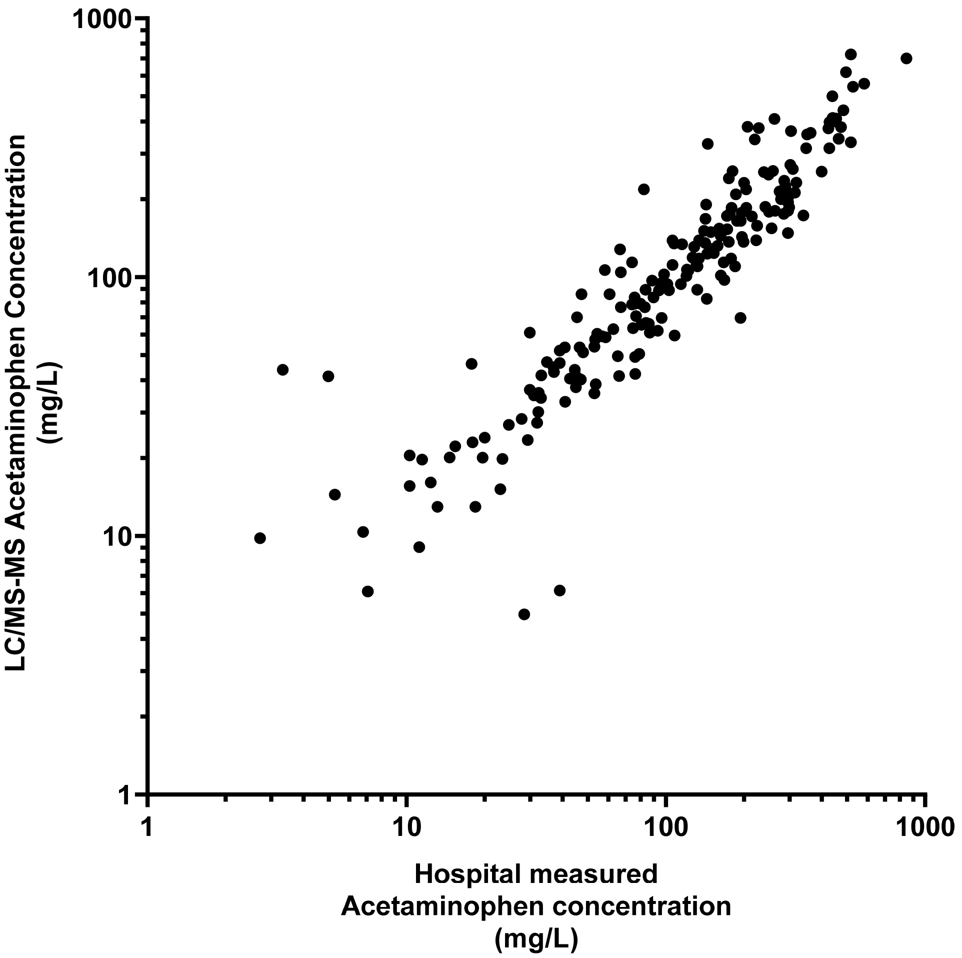

Figure S1: LCMS measured vs hospital measured acetaminophen concentrations (n=195).

Table S1: Outcome hepatotoxicity, only those patients with an ALT < 1000 U/L on presentation and not receiving acetylcysteine at the time of the initial acetaminophen and acetaminophen metabolite measurements

Metabolites/ Biomarker with initial acetaminophen concentration	No hepatotoxicity (median) (IQR, n)	Hepatotoxicity (median) (IQR, n)	p value	AUC ROC curve (95% CI)
APAP-hospital lab (µmol/L)	702<br>(301 - 1353, n=119)	1106<br>(453 - 1910, n = 21)	NS	0.59<br>(0.45 to 0.72)
ALT	20<br>(15 - 28, n=117)	133<br>(57 – 221, n= 21)	<0.0001	0.90<br>(0.81 to 0.98)
Acetaminophen Ratio	1.19<br>(0.43 – 2.01, n= 119)	3.45<br>(3.45 – 7.13, n=21)	<0.0001	0.82<br>(0.72 – 0.93)
**Acetaminophen metabolite**				
APAP-LC/MS (µmol/L)	646<br>(297 – 1145, n= 119)	867<br>(360 – 1460, n= 21)	NS	0.57<br>(0.43 to 0.71)
APAP-Glu (µmol/L)	461<br>(299 – 776, n=119)	568<br>(410 – 1120, n=21)	NS	0.61<br>(0.47 – 0.75)
APAP-Sul (µmol/L)	72.4<br>(50.4 – 102.5, n=119)	48.9<br>(24.7 – 72.2, n = 21)	0.0031	0.70<br>(0.58 to 0.82)
APAP-Cys (µmol/L)	9.4<br>(5.9 - 16.8, n=119)	37.1<br>(26.0 – 52.3, n=21)	<0.0001	0.87<br>(0.77 to 0.99)
APAP-Mer (µmol/L)	1.03<br>(0.61 – 1.74, n= 119)	2.26<br>(1.05 – 4.11, n= 21)	0.0023	0.71<br>(0.57 to 0.84)
CYP total	10.3<br>(6.8 – 18.3, n=119)	39.9<br>(27.8 – 55.3, n=21)	<0.0001	0.87<br>(0.76 to 0.98)
Total Metabolites (µmol/L)	545<br>(388 – 906, n=119)	654<br>(502 – 1237, n=21)	NS	0.60<br>(0.46 – 0.74)
% APAP-Glu	85<br>(79 – 89, n=119)	89<br>(82 – 91, n=21)	NS	0.61<br>(0.47 to 0.74)
% APAP-Sul	12.9<br>(8.5 – 18.2, n=119)	5.8<br>(4.6 – 9.9, n=21)	<0.0001	0.78<br>(0.66 to 0.91)
% CYP metabolites	1.64<br>(1.16 – 2.80, n= 119)	5.55<br>(4.09 – 7.89, n= 21)	<0.0001	0.85<br>(0.76 to 0.94)
**Proposed Risk Stratification Tools**				
APAP-Cys/APAP-Sul	0.12<br>(0.07 – 0.23, n = 119)	0.88<br>(0.63 – 1.08, n = 21)	<0.0001	0.91<br>(0.83 to 0.98)
ALT * APAP-Cys/APAP-Sul	2.65<br>(1.12 – 5.12, n=119)	134<br>(42.2 – 237, n = 21)	<0.0001	0.94<br>(0.88 to 1.00)
Acetaminophen Ratio*APAP-Cys	11.7<br>(2.33 – 22.5, n= 119)	110.1<br>(33.0 – 370, n= 21)	<0.0001	0.88<br>(0.79 – 0.97)
Acetaminophen Ratio*APAP-Cys*ALT	190<br>(58.0 – 525, n= 117)	16392<br>(1887 – 72202, n= 21)	<0.0001	0.95<br>(0.91 to 0.99)

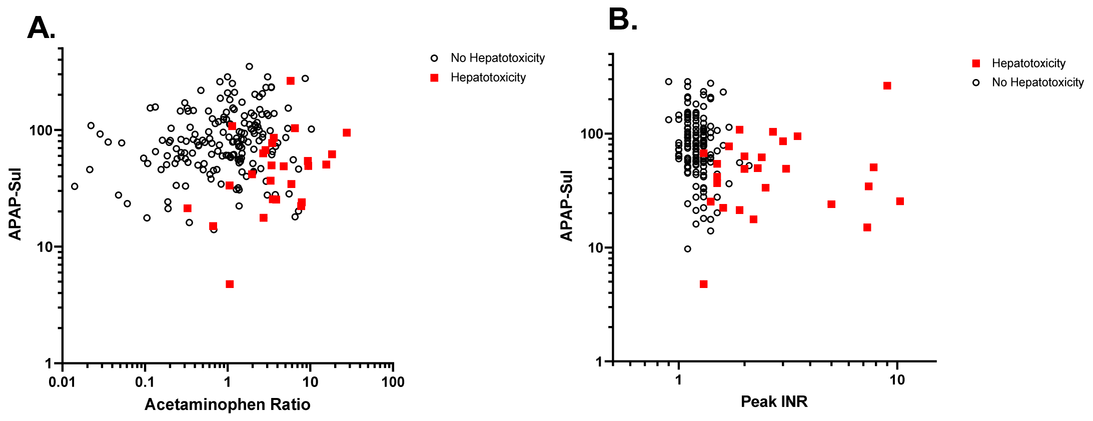

Figure S2: Correlation between A/ APAP-Sul and acetaminophen ratio (n= 200) B/ APAP-Sul and peak INR (n= 165)
